# Novel single nucleotide mutations in exon-10 of human coagulation Factor V gene in patients with pulmonary thromboembolism

**DOI:** 10.34172/jcvtr.2020.02

**Published:** 2020-01-28

**Authors:** Latheef Kasala, Rajasekhar Durgaprasad, Vanajakshamma Velam

**Affiliations:** Department of Cardiology, Sri Venkateswara Institute of Medical Sciences & University, Tirupati, Andhra Pradesh, India

**Keywords:** Pulmonary Thromboembolism, Factor V, Mutations

## Abstract

***Introduction:*** Acute pulmonary thromboembolism (PTE) presents with wide spectrum and has variable prognosis. Factor V Leiden (FVL) is the most common inherited thrombophilia, with a prevalence of 3%-7% in the general US population, approximately 5% in Whites, 2.2% in Hispanics and 1.2% in Blacks. PTE most commonly originates from venous thrombosis. The occurrence of venous thromboembolism is a culmination of environmental and genetic risk factors. The current study was sought to identify the mutations in exon-10 of FV gene in patients with PTE.

***Methods:*** Sixty cases diagnosed with PTE and 50 healthy controls were enrolled in the present study. Mutation studies in exon-10 of Factor V gene included PCR-DNA sequencing method.

***Results:*** Of 60 patients, we found two novel transition type point mutations: c.1538 G>A and c.1601 G>A in exon-10 of Factor V which is responsible for the cleavage site for aPC. These point mutations resulted in single amino acid change in protein sequence at p.Arg513Lys and p.Arg534Gln respectively. These mutations prevent efficient inactivation of Factor V and Factor V remains active which facilitates over production of thrombin leading to generation of excess fibrin and excess coagulation which results in deep vein thrombosis and PTE.

*** Conclusion:*** We report two novel point mutations (c.1538 G>A and c.1601 G>A) in exon-10 of Factor V gene in Indian patients with PTE.

## Introduction

The presentation of acute pulmonary thromboembolism (PTE) has wide spectrum and variable prognosis. The most common inherited thrombophilia is Factor V Leiden (FVL), with prevalence rates of 3%-7%, 5%, 2.2% and 1.2% in general US population,^1^ Whites, Hispanics and in Blacks respectively.^2^ However, the prevalence rate of FVL in patients with venous thromboembolism is 50%.^1,3^ The incidence of venous thromboembolism in patients with FVL is less i.e., 0.5% per annum.^4,5^

In maintaining homeostasis between coagulation and anticoagulation pathways Factor V (FV) has a critical position. FVL consequences in a hypercoagulable state by both uprising coagulation and reducing anticoagulation. This mutation causes FV resistant to activated protein C (aPC) which can prevent cleavage and inactivation of FV, a condition acknowledged as aPC resistance. As a result, more factor Va is available inside the prothrombinase complex, increasing coagulation by amplified production of thrombin.^6-8^

Furthermore, a cofactor produced by cleavage of FV at 506 position is believed to support aPC in destroying factor VIIIa, along with protein S. Thus, people with FVL have less anticoagulant activity from aPC due to lack of this cleavage product. For hypercoagulable state of FVL associated aPC resistance, both amplified coagulation and reduced anticoagulation are appeared to contribute equally.^9-11^

In view of importance of FV mutations in the pathogenesis of PTE, the current study was purposed to identify the mutations in exon-10 of FV gene in patients with PTE.

## Materials and Methods

This is a prospective, non-interventional, case-control study conducted in the department of Cardiology, Sri Venkateswara Institute of Medical Sciences (SVIMS), Tirupati between May 2013 and December 2015.

### Inclusion criteria

All the prospectivepatients with confirmed acute PTE on computed tomography pulmonary angiography (CTPA) were enrolled into the study group.

### Exclusion criteria

Patients with renal impairment, preexisting chronic lung disorders and not willing to give consent were excluded.

### Patients

Sixty prospective patients diagnosed with acute PTE [confirmed on CTPA] and admitted in the department of Cardiology, SVIMS for treatment.

### Controls

Fifty healthy voluntary subjects without any documented signs and symptoms for PTE and other coagulable disease were studied for single nucleotide mutations in exon-10 of FV.

On admission patients were assessed for medical history, clinical presentation, risk factors, vitals, serum creatinine. All the patients underwent electrocardiogram, echocardiogram and lower limb venous ultrasound.

### Genetic analysis

Five milliliters of peripheral venous blood sample was collected from all the study patients. QIAamp DNA Mini spin-column [Qiagen] DNA extraction kit was used for the isolation of genomic DNA from the blood samples, and the extracted samples were analyzed on 1% agarose gel through electrophoresis method.^12,13^

### Polymerase chain reaction procedure

Polymerase chain reaction (PCR) amplification was done using Eppendorf Mastercycler nexus gradient-flexlid model, Hamburg, Germany. The following Oligonucleotide primers were designed using Oligo-6, NCBI blast and Primer-3 software packages and synthesized at Eurofins genomics India Pvt. Ltd. Bengaluru, India (see Table 1).^14^ PCR reaction conditions are summarized in Table 2.

**Table 1:** Details of Oligonucleotide primers & PCR reaction mixture

**Oligonucleotide Primers**	Forward Primer: 5’-ACCCACAGAAAATGATGCCCAG-3’ Reverse Primer: 5’-TGCCCCATTATTTAGCCAGGAG-3’
**PCR reaction mixture**	A total volume of 50 µL reaction mixture comprising of the below components was prepared in Milli Q water- 1x assay buffer, 1.5 mmol MgCl_2_, 500 ng template DNA, 50 pico mole forward primer, 50 pico mole reverse primer, 100 µmol dNTPs mix and 1U of Taq polymerase.

MgCl_2_: magnesium chloride; µL: micro liter; mmol: milli mole; ng: nanogram; µmol: micro mole; dNTP: deoxy-ribo nucleotide triphosphate; U: unit; Taq: *Thermasaquaticus*.

**Table 2:** PCR reaction conditions

**Phase of PCR**	**Temperature**	**Duration**	**No. of Cycles**
Initial denaturation	94°C	10 minutes	35 cycles
Denaturation	94°C	60 seconds	
Annealing	61°C	45 seconds	
Extension	72°C	60 seconds	
Final extension	72°C	10 minutes	

PCR, polymerase chain reaction.

The amplified PCR products were analyzed on 2% agarose gel in 1X TAE [Tris-Acetate-EDTA, pH: 7.8] to confirm the targeted amplification.^12^ The purification of PCR products resolved in 2% agarose gel was done by electro elution method with NucleoSpin^®^ PCR (NP-PCR) Purification kit (Taurus Scientific, USA).^12^ The amplified PCR products were sequenced at Eurofins Genomics India Pvt Ltd., Bengaluru, India.

### Sequence analysis

**Figure-1:**
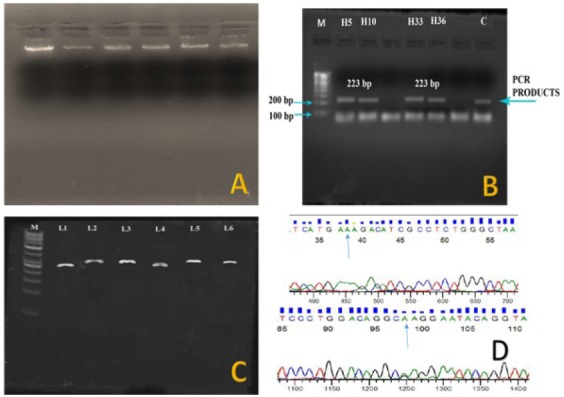
Genetic analysis of exon-10 of Factor V gene. 1% agarose gel showing isolated pure genomic DNA. **(B)**2% agarose gel showing 223 bp PCR product of exon-10 of FV gene. Lane M: 100 bp DNA marker; Lane H5, H10, H33, H36: PCR amplicons of patients; C: PCR amplicon of Control. **(C)** SSCP analysis of exon-10 of FV gene PCR amplicons, Lane M: 100 bp DNA marker, Lane L1: wild type, Lane L2-L6 mutated exon-10 of Factor V gene. **(D)** Chromatogram showing single nucleotide mutations in 2 sequences.

Multiple sequence alignment was performed using ClustalX tool(Version 1.83, National Center for Biotechnology Information, Bethesda, MD) to compare the sequences and the mutations were noted. Nucleotide sequences were translated into amino acid sequences using Expert Protein Analysis System (ExPASy) analysis and the changes in amino acid sequences were noted for each sequence. Schematic representation of various steps of genetic analysis is shown in Figure 1.

### Structural analysis

#### Structural superimposition of control and mutated FV structure

The comparative structural prediction of control and mutated FV structures was performed to ascertain the variations in the domain and non-domain regions of structure. An alignment of superimposed structures and similarities were predicted by root mean square deviation (RMSD) values. PyMOL was used to study the positional variations in alpha helix, beta sheets, interacting residues and active sites.

### Statistical analysis

Data was collected on MS-Excel spread sheets. Mean with standard deviation (SD) and frequencies with percentages were calculated for continuous and categorical data. SPSS version 20.0 (IBM Corp., Armonk, NY: USA) was used for the analysis.

## Results

Sixty consecutive acute PTE patients were studied. Baseline characteristics of the study population are shown in Table 3. Mean age of the study patients was 41.2±12.9 years (range: 21-76 years). Forty-eight (80%) were male and 12 (20%) were female. The principle symptoms were dyspnea (100%), chest pain (33.3%), syncope (20%), hemoptysis (16.7%), and altered sensorium (3.3%). Frequencies of different risk factors were - 43.3% dyslipidemia, 30% smoking, 6.7% cancer, 13.3% hypertension, 10% diabetes, 3.3% stroke and 6.7% coronary artery disease. Mean heart rate was 118±15 beats per minute. Mean systolic blood pressure (SBP) was 114.3 ± 16.6 mm Hg and diastolic blood pressure (DBP) was 73.2 ± 9.3 mm Hg.

**Table 3:** Baseline characteristics of study population

**Characteristic**	**Study group (n=60)**	**Control group (n=50)**	***P*** **value**
Age (y)	41.2 ± 12.9	40.5 ± 10.3	NS
Male	48 (80%)	40 (80%)	NS
Chest Pain	20 (33.3%)	00	NA
Dyspnea			NA
NYHA Class-1	2 (3.3%)	-	
NYHA Class-2	14 (23.3%)	-	
NYHA Class-3	26 (43.3%)	-	
NYHA Class-4	18 (30.0%)	-	
Cancer	4 (6.7%)	00	NA
Stroke	2 (3.3%)	00	NA
CAD	4 (6.7%)	00	NA
Dyslipidemia	26 (43.3%)	00	NA
Smoking	18 (30%)	00	NA
Hypertension	8 (13.3%)	00	NA
Diabetes	6 (10%)	00	NA
HR, bpm	118.5 ± 15.6	119.4 ± 11.8	NS
SBP, mm Hg	114.3 ± 16.6	111.1 ± 14.3	NS
DBP, mm Hg	73.2 ± 9.3	75.6 ± 7.8	NS

NYHA: New York Heart Association; CAD: coronary artery disease; HR: heart rate; SBP: systolic blood pressure; DBP: diastolic blood pressure, NS: not significant; NA: not applicable.

**Figure 2:**
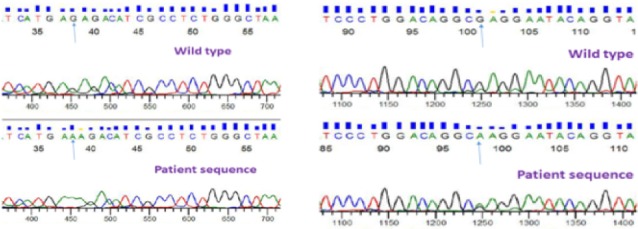
Comparison of chromatograms: patient sequences vs wild type sequence.

We found two novel transition type point mutations: c.1538 G>A and c.1601 G>A in exon-10 of FV which is responsible for the cleavage site for aPC (Figure 2). ExPASy analysis revealed that these point mutations ensued a change of single amino acid in protein sequence at p.Arg513Lys and p.Arg534Gln, respectively. We have not find any mutation in exon-10 of FV among healthy controls.

### Structural analysis

**Figure 3:**
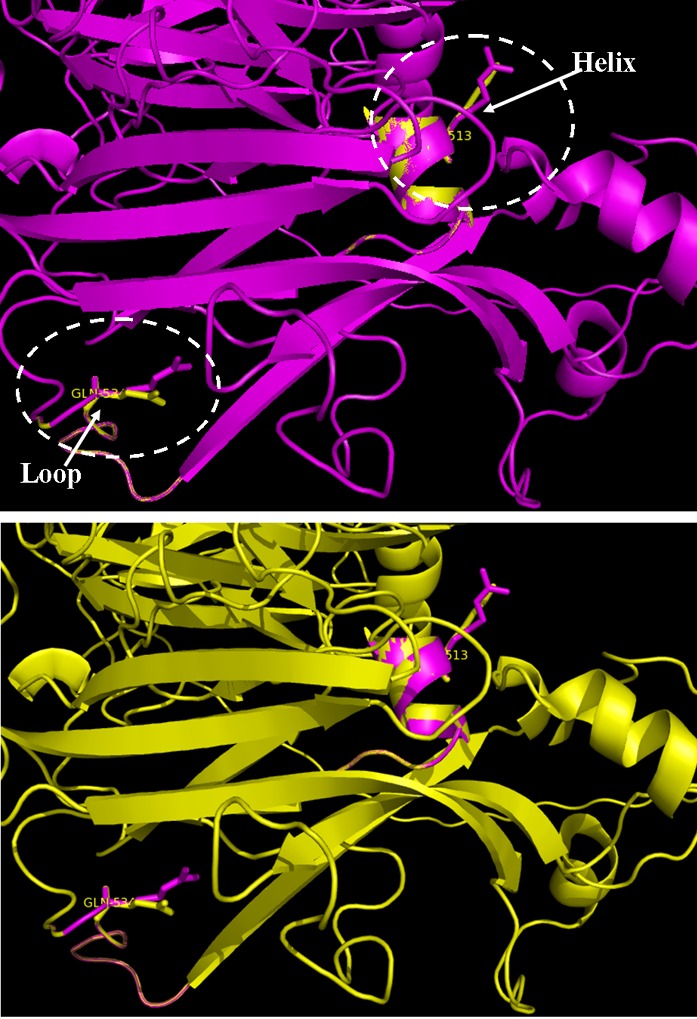
Structural analysis of FV gene.
**Top:** PyMOL view of wild-type FV structure (Pink color), **Bottom:**Superimposed conformations of wild- type (Yellow color) with mutated p.R513K and p.R534Q structure showing RMSD 0.223Å.

The built mutant FV structure when superimposed with the control FV structure, exhibited variations with an RMSD values in both domain and non-domain regions, with variable number of strands, helices, helix–helix interactions, β-α-β units, hairpins, β-bulges, β-turns, and γ-turns which can explain the major conformational changes in the mutant FV structure (Figure 3). Mutations in FV gene causes Factor Va resistant to aPC degradation, that can further increase the risk of venous thromboembolism in these patients.

## Discussion

FVL and prothrombin mutations, among the several, are thought to be the most frequent causative factors for inherited thrombophilia. However, contribution of these factors varies from population to population.

Factor Va and factor VIIIa were cleaved at conserved arginine (R) residues by aPC to inhibit coagulation at positions R306, R506, and less importantly, at R679.

FVL is a missense mutation in the FV gene at position G1691A, which can result in change of amino acids from arginine to glutamine (R506Q)^7,15,16^ that slows the inactivation of factor Va by aPC and thus producing a genetic risk factor in association with environmental risk factors which causes an increased risk for venous thrombosis.

Other, less common FV mutations also affect aPC resistance, with differing prothrombotic risks. Of these, one of the more important is FV_R2_ (H1299R) which is tightly associated to several other polymorphisms and collectively named the R2 haplotype.^17^

FV_Liverpool_ (I359T), another mutation, which has been reported in a family in which it was alone asymptomatic, but the same with mutation on the other FV allele (a premature stop codon) was showed association with low FV levels, aPC resistance, and increased thrombotic risk.^18^ FV_Cambridge_ (R306T) and FV_Hong Kong_ (R306G) are the two other rare mutations which exhibit insignificant aPC resistance and slightly reduced aPC cofactor activity in vitro. However, no association of R306 mutations with increased risk of thrombosis was exhibited in vivo.^19,20^

FVL prevalence in Indian population is variable from north to south and low occurrence of FVL was reported in northern states. Whereas, its relevance in southern part is insignificant. In concurrence with the findings reported by Himabindu et al^21^ in the present study we have not find FVL mutation in our study cohort.

In the current study, we found two novel transition type point mutations, i.e., c.1538 G>A and c.1601 G>A in exon-10 of FV which are responsible for the cleavage site for aPC. These point mutations resulted in single amino acid change in protein sequence at p.Arg513Lys and p.Arg534Gln respectively.

These mutations may prevent efficient inactivation of FV by aPC and remains active which facilitates over production of thrombin leading to generation of excess fibrin and excess clotting which results in DVT and PTE. The present study findings require further validation with larger sample size to ascertain the precise impact of these mutations in pathophysiology of PTE.

### Limitations

This is a single center study and only South-Indian ethnic population was included. Further multi-centric studies with large sample size and different ethnic populations are required to confirm these findings. The methods used for DNA sequencing in the present study are relatively time-consuming and expensive.

## Conclusion

We report two novel point mutations (c.1538 G>A and c.1601 G>A) in exon-10 of Factor V gene in Indian patients with PTE. Occurrence of novel insertional mutations G>A at 1538 and 1601 in exon 10 of FV gene which were identified in this study may not be significant. However, if the same study is extended to a larger population may reveal new insights into thromboembolic factors in PTE.

## Competing interests

None.

## Ethical approval

This study was approved by the Institutional Ethics Committee of our institute with IEC No. 21.

## Funding sources

None.

## Acknowledgments

We would like to thank Dr. Usha Kalawat, Nodal Officer, ICMR-VRDL, Department of Microbiology, SVIMS, Tirupati for permitting us to utilize PCR services.
